# Co-Infection of Blacklegged Ticks with *Babesia microti* and *Borrelia burgdorferi* Is Higher than Expected and Acquired from Small Mammal Hosts

**DOI:** 10.1371/journal.pone.0099348

**Published:** 2014-06-18

**Authors:** Michelle H. Hersh, Richard S. Ostfeld, Diana J. McHenry, Michael Tibbetts, Jesse L. Brunner, Mary E. Killilea, Kathleen LoGiudice, Kenneth A. Schmidt, Felicia Keesing

**Affiliations:** 1 Program in Biology, Bard College, Annandale-on-Hudson, New York, United States of America; 2 Cary Institute of Ecosystem Studies, Millbrook, New York, United States of America; 3 School of Biological Sciences, Washington State University, Pullman, Washington, United States of America; 4 Department of Environmental Studies, New York University, New York, New York, United States of America; 5 Department of Biology, Union College, Schenectady, New York, United States of America; 6 Department of Biological Sciences, Texas Tech University, Lubbock, Texas, United States of America; University of Minnesota, United States of America

## Abstract

Humans in the northeastern and midwestern United States are at increasing risk of acquiring tickborne diseases – not only Lyme disease, but also two emerging diseases, human granulocytic anaplasmosis and human babesiosis. Co-infection with two or more of these pathogens can increase the severity of health impacts. The risk of co-infection is intensified by the ecology of these three diseases because all three pathogens (*Borrelia burgdorferi, Anaplasma phagocytophilum*, and *Babesia microti*) are transmitted by the same vector, blacklegged ticks (*Ixodes scapularis*), and are carried by many of the same reservoir hosts. The risk of exposure to multiple pathogens from a single tick bite and the sources of co-infected ticks are not well understood. In this study, we quantify the risk of co-infection by measuring infection prevalence in 4,368 questing nymphs throughout an endemic region for all three diseases (Dutchess County, NY) to determine if co-infections occur at frequencies other than predicted by independent assortment of pathogens. Further, we identify sources of co-infection by quantifying rates of co-infection on 3,275 larval ticks fed on known hosts. We find significant deviations of levels of co-infection in questing nymphs, most notably 83% more co-infection with *Babesia microti* and *Borrelia burgdorferi* than predicted by chance alone. Further, this pattern of increased co-infection was observed in larval ticks that fed on small mammal hosts, but not on meso-mammal, sciurid, or avian hosts. Co-infections involving *A. phagocytophilum* were less common, and fewer co-infections of *A. phagocytophilum* and *B. microti* than predicted by chance were observed in both questing nymphs and larvae fed on small mammals. Medical practitioners should be aware of the elevated risk of *B. microti*/*B. burgdorferi* co-infection.

## Introduction

Co-infections from tickborne diseases are a threat to human health in the northeastern and midwestern United States, but the risk of acquiring a co-infection is not fully understood. Lyme disease, caused by the spirochete pathogen *Borrelia burgdorferi*, is an established public health problem in the United States, with >25,000 reported cases annually from 2008–2011 (CDC, 2013). Annual cases of human granulocytic anaplasmosis, caused by the gram-negative intracellular bacterium *Anaplasma phagocytophilum*, have been increasing in the last decade [1]. Human babesiosis, caused by the protozoan blood parasite *Babesia microti*, has also been increasing in prevalence, especially in the northeastern United States [2]–[4]. These three tickborne pathogens – *A. phagocytophilum, B. microti*, and *B. burgdorferi* – are transmitted by the same vector, *Ixodes scapularis*, the blacklegged tick, with the great majority of human cases transmitted by the nymphal stage of these ticks [5]. *I. scapularis* ticks can be infected with any combination of these pathogens or all three simultaneously [6]. The risk to humans of acquiring co-infection depends on both their exposure to tick bites and the infection status of the ticks.

Co-infection of multiple tickborne pathogens can affect the intensity and duration of symptoms in humans, and make diagnosis and treatment more challenging. Co-infection of *Babesia microti* and *Borrelia burgdorferi* has been the most frequently observed human co-infection in several studies of regions in which all three pathogens are endemic [7], [8]. *B. microti*/*B. burgdorferi* co-infection can cause more severe or persistent symptoms in human patients [7], [9]–[13] (but see also [14], [15]). Humans could in theory become co-infected either through the bite of a single co-infected tick, or sequential bites of ticks each transmitting a different pathogen; in this study we focus on the risk of exposure to multiple pathogens that arises from bites of co-infected ticks. Rates of transmission from infected ticks to vertebrate hosts can vary with co-infection (e.g. [16]).

The risk of exposure to more than one pathogen from a single bite of a co-infected tick depends on both: (1) the prevalence of co-infections in questing nymphs, and (2) the prevalence of co-infections in the wildlife hosts these ticks feed on as larvae. As none of these pathogens are known to be vertically transmitted [6], co-infected questing nymphs must have obtained multiple infections from feeding on a co-infected host as larvae. Few consistent patterns have emerged from observations of co-infection in questing ticks. Since larval *I. scapularis* ticks typically only have a single blood meal, co-infected nymphal ticks are likely a result of larval ticks feeding on co-infected hosts. Pathogens interacting within a single host could in theory facilitate one another, directly or indirectly compete, or have no additive effects [17], evidenced by positive, negative, or neutral relationships in pathogen infection status or abundance within hosts. Negative, positive, and neutral relationships of pathogen occurrence in both nymphal and adult questing ticks have been reported [18]. We focus this study on nymphs as this stage is responsible for the majority of human infections with tick-borne disease [5]. Co-infection studies to date have focused on either questing ticks or a few reservoir hosts, but have neglected simultaneous assessment of co-infection frequencies in both questing nymphs and the wildlife hosts from which they acquire pathogens.

In wildlife hosts, co-infection studies on tickborne pathogens include both observational studies based on serology and experimental studies on laboratory animals. Within a host, multiple parasite infections can be modulated by host immune responses, priority effects, and interactions among pathogens [17], [19]. In a long-term study of field voles (*Microtus agrestis*), evidence for both positive and negative interactions between *B. microti* and *A. phagocytophilum* was documented, with the outcome dependent on the duration of *A. phagocytophilum* infection [20]. Experimentally, independent transmission of *B. burgdorferi* and *A. phagocytophilum* both to and from *I. scapularis* ticks has been demonstrated [16]. However in white-footed mouse (*Peromyscus leucopus*) hosts, prior infection with either pathogen inhibits establishment of the second [21], reducing the likelihood of co-transmission to ticks. In contrast, prior ecological research on associations between vertebrate hosts and these three zoonotic pathogens suggests that co-infection in ticks could be common. Certain host species, such as *P. leucopus*, have high reservoir competence (probability of transmitting infection to uninfected ticks) for all three pathogens [22]–[24], potentially facilitating tick co-infection. In addition, 45% of 463 antibody-positive wild white-footed mice sampled in Connecticut were shown to be seropositive for all three pathogens [25], suggesting high exposure rates. These conflicting results yield limited predictive power concerning co-infection patterns and an incomplete understanding of underlying processes.

In this study, we sought to improve our understanding of the pattern and processes of co-infection. Our first aim was to quantify patterns of co-infection of *A. phagocytophilum, B. microti*, and *B. burgdorferi* in questing *I. scapularis* nymphs, given their importance in human infections [5]. Our second aim was to determine whether co-infection in questing nymphs was caused by transmission biases within groups of hosts. To accomplish this, we surveyed both questing nymphal ticks and newly molted nymphs fed as larvae on specific host species (hereafter ‘host-collected ticks’) in an area endemic to all three pathogens (Dutchess County, NY, USA). Our general strategy was to assess infection status of: (1) questing nymphs sampled from many different landscape contexts likely representing different vertebrate host communities; and (2) host-collected ticks from known mammalian and avian hosts. This allowed us to determine whether co-infection rates were different from what would be predicted if pathogens were assorting independently and to assess which hosts might be responsible for deviations from independent assortment.

## Materials and Methods

### Ethics statement

All animal care and husbandry was conducted with approval from the Cary Institute of Ecosystem Studies Institutional Animal Care and Use Committee (IACUC). We worked under Cary IACUC protocols 06-03 and 09-01. The New York State Department of Environmental Conservation was the permitting authority for animal use, and our approval was through LCP 639. We followed the guidelines for the care and use of animals of the American Society of Mammalogists [26] and the National Research Council [27]. Vertebrate animals were trapped on private land owned by the Cary Institute of Ecosystem Studies.

Questing nymphal ticks were collected on 187 field sites throughout Dutchess County (Figure S1). 122 sites were on private land from which landowner permission was obtained for tick sampling. Richard S. Ostfeld (ostfeldr@caryinstitute.org) is the contact person for further information regarding sampling locations. For the remaining 65 sites, permission for tick sampling was obtained from the following entities: City of Poughkeepsie, NY (3 sites), Dutchess County Parks Department (3 sites), Hyde Park Central School District (1 site), National Park Service (10 sites), National Park Service/The Nature Conservancy (1 site), New York City Department of Environmental Protection (3 sites), New York State Department of Environmental Conservation (7 sites), New York State Office of Parks, Recreation, and Historic Preservation (16 sites), New York State Department of Environmental Conservation/New York State Office of Parks, Recreation, and Historic Preservation (5 sites), Putnam Highlands Audubon Society (1 site), Red Hook Central School District (1 site), Scenic Hudson Land Trust (1 site), The Nature Conservancy (6 sites), Town of Beekman, NY (1 site), Town of Dover, NY (1 site), Town of Fishkill, NY (2 sites), Town of LaGrange, NY (1 site), Town of Union Vale, NY (1 site), and the Winnakee Land Trust (1 site). No studies involved endangered or protected species.

### Tick collection and molecular analysis

Questing nymphal ticks were collected from 161 field sites distributed throughout Dutchess County, NY, USA, in 2011 and 2012. Sites were selected using a geographic information systems (GIS) map of forested and non-forested land cover digitized from aerial orthophotos generated in 2009. An initial candidate list of 2,500 random points was generated using a random point overlay. These points were then stratified by the percentage of forest cover in the surrounding landscape, to provide equal representation along a gradient of forest cover, from extensively forested to highly fragmented. Sites were eliminated when access was poor or property owners could not be located or recruited, resulting in a group of 187 sites that were sampled. Ticks were collected by dragging a 1 m^2^ corduroy cloth along 400 m transects at each site once or twice during peak nymphal activity (early summer). Any site that yielded fewer than 10 nymphal ticks over the two years of sampling was eliminated from further analysis (26 sites). Of the remaining 161 sites, 83 were sampled in 2011 only, 11 were sampled in 2012 only, and 67 were sampled in both years. An average of 27.1 ticks (SD: 12.7, range: 10–63 ticks) were sampled per site.

To obtain ticks known to have fed on specific host species (‘host-collected ticks’), wildlife hosts were trapped during the summers of 2008, 2009, and 2010 during the peak of larval activity on the property of the Cary Institute of Ecosystem Studies in Millbrook, NY, USA. Host species included four small mammal species: Northern short-tailed shrews (*Blarina brevicauda*), white-footed mice (*Peromyscus leucopus*), masked shrews (*Sorex cinereus*), and eastern chipmunks (*Tamias striatus*); two meso-mammal species: Virginia opossums (*Didelphis virginiana*) and raccoons (*Procyon lotor*); three sciurid species: Southern flying squirrels (*Glaucomys volans*), Eastern gray squirrels (*Sciurus carolinensis*), and North American red squirrels (*Tamiasciurus hudsonicus*); and four bird species: veeries (*Catharus fuscescens*), gray catbirds (*Dumetella carolinensis*), wood thrushes (*Hylocichla mustelina*), and American robins (*Turdus migratorius*). Division into these functional/taxonomic groups was based on prior research suggesting disparate roles of each group in feeding and infecting blacklegged ticks [28]–[30]. Hosts were held in the Cary Institute Rearing Facility for 3–8 days in cages with wire mesh floors. Engorged larvae were collected during daily inspections of pans containing multiple layers of moistened paper towels beneath each cage. After collection of engorged larvae, hosts were returned to their point of capture. Some hosts with low natural body burdens were infested with unfed larval ticks as described in Keesing et al. [29]. Engorged larval ticks were held in moistened plaster vials until molting to the nymphal stage was completed. Individual hosts were included only if they produced at least 10 newly molted nymphs, with the exception of three species with low body burdens: *G. volans, S. cinereus*, and *T. migratorius*. Realized reservoir competence was calculated as the average percent larval ticks infected by an individual host of a given species. For a full description of sampling for larval ticks from hosts, see Hersh et al. [22].

All sampled ticks were flash frozen in liquid nitrogen and stored at −80°C for subsequent molecular analysis. Total genomic DNA was extracted from individual frozen ticks using a Qiagen DNeasy Blood and Tissue kit (Qiagen, Hilden, Germany) or a Gentra PureGene Tissue Kit (Qiagen). *A. phagocytophilum, B. microti*, and *B. burgdorferi* infections in ticks were detected using real-time PCR. *A. phagocytophilum* and *B. burgdorferi* infections were detected in a multiplex reaction [23], [31]. *A. phagocytophilum* was detected using primers ApMSP2f (5′-ATG GAA GGT AGT GTT GGT TAT GGT ATT-3′) and ApMSP2r (5′-TTG GTC TTG AAG CGC TCG TA-3′) and TaqMan probe (Life Technologies) ApMSP2p (5′-VIC-TGG TGC CAG GGT TGA GCT TGA GAT TG-TAMRA-3′) targeting the *msp2* gene. *B. burgdorferi* was detected using primers Bb23Sf (5′-CGA GTC TTA AAA GGG CGA TTT AGT-3′) and Bb23Sr (5′-GCT TCA GCC TGG CCA TAA ATA G-3′) and TaqMan probe Bb23Sp (5′-6FAM-AGA TGT GGT AGA CCC GAA GCC GAG TG-TAMRA-3′) targeting the 23S ribosomal RNA (rRNA) gene. *B. microti* infection was detected using a singleplex reaction following Hersh et al. [22] using primers smbaJF (5′-GCG TTC ATA AAA CGC AAG GAA GTG T-3′) and smbaKR (5′ –TGT AAG ATT ACC CGG ACC CGA CG-3′) and a SYBR green probe targeting the 18S rRNA gene.

### Data Analysis

A permutation test was developed to determine if co-infection occurred more or less frequently than expected by chance. Observed frequencies of all three pathogens were resampled 100,000 times independently and without replacement. Next, the proportion of samples in which the difference between the observed prevalence of each infection type and the permutation mean was as or more extreme than the difference between the permutation mean and each permuted sample was calculated, such that *p*  =  (number of samples in which |(permutation mean – observed data)| ≥ |(permutation mean- permutation data point)| +1)/(number of permutations +1) [32]. An effect size was then quantified as the ratio of the observed level of co-infection to the permutation mean. This test was performed separately for questing nymphs and host-collected ticks. In the case of host-collected ticks, data for each species were resampled individually using the same permutation analysis if over 100 ticks were collected from that species. Permutation tests were also run for broader taxonomic groups (small mammals, meso-mammals, sciurids, and birds, as above) using a nested analysis in which each species in the group was resampled separately, and resampled frequencies were combined to calculate *p*-values. All analyses were carried out in the statistical software package R 3.0.1 (The R Foundation for Statistical Computing, www.R-project.org)

## Results

In total, 7,643 ticks were sampled for infection with *Borrelia burgdorferi* (Bb), *Anaplasma phagocytophilum* (Ap), and *Babesia microti* (Bm). Of these ticks, 4,368 questing nymphs were sampled from 2011–2012 from 161 sites across Dutchess County, while 3,275 host-collected ticks were sampled from 181 wildlife host individuals from 2008–2010.

### Questing nymphs

**Figure 1:**
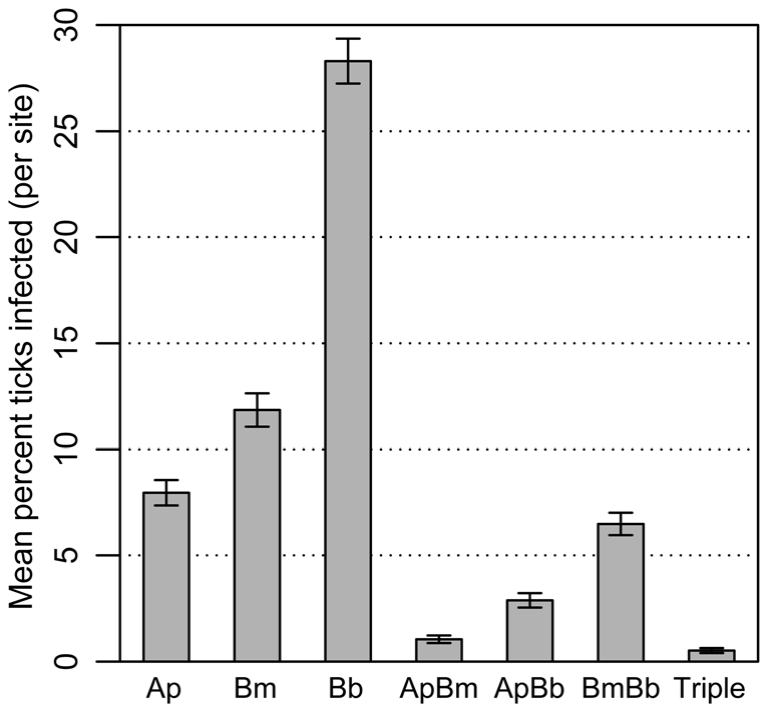
Mean levels of co-infection prevalence of *Anaplasma phagocytophilum, Babesia microti*, and *Borrelia burgdorferi* in questing *Ixodes scapularis* nymphs, by site. Mean levels of co-infection with *Anaplasma phagocytophilum* (Ap), *Babesia microti* (Bm), and *Borrelia burgdorferi* (Bb) in questing nymphal *Ixodes scapularis* ticks by site, 2011–2012. Each category represents mean overall prevalence as opposed to the prevalence of each specific infection type – for example, the “Ap” bar represents not just single infections but also ticks co-infected with Ap and either or both of the other two pathogens. Error bars represent standard error. For individual years see Figure S2.

Co-infection of *B. microti* and *B. burgdorferi* was commonly observed in questing nymphs (Figure 1, Figure S2). The mean prevalence of dual *B. microti*/*B. burgdorferi* infection per site was 5.96% (Figure 1), while the overall prevalence of all ticks sampled was 6.68% (292 of 4,368 nymphs). Of single infections, *B. burgdorferi* was the most common and *A. phagocytophilum* was the most rare (Figure 1). Co-infection combinations involving *A. phagocytophilum* were less common than *B. microti*/*B. burgdorferi* co-infection (Figure 1, Figure S2). Mean prevalence per site of *A. phagocytophilum/B. microti* and *A. phagocytophilum/B. burgdorferi* were 0.53% and 2.35%, respectively (Figure 1). Triple infections were uncommon, occurring at 0.52% prevalence per site.

**Figure 2:**
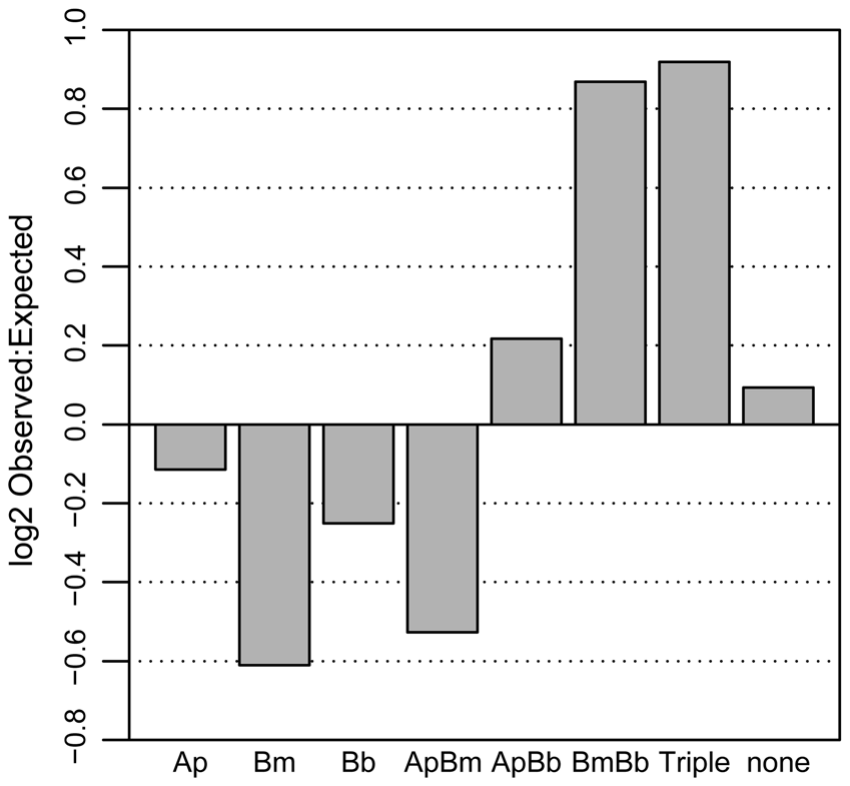
Log (base 2) of the observed: expected ratio of questing *Ixodes scapularis* nymphs. Log (base 2) of observed: expected ratio of each infection status of questing *Ixodes scapularis* ticks sampled at 161 sites across Dutchess County, NY, USA. The magnitude and direction of the log ratios illustrates the extent to which the observed levels of co-infection differed from expected levels of co-infection due to random assortment of pathogens. Pathogens sampled include *Anaplasma phagocytophilum* (Ap), *Babesia microti* (Bm), and *Borrelia burgdorferi* (Bb). Expected infection frequencies are based on 100,000 random permutations of infection frequencies for each pathogen.

Observed levels of co-infection frequently deviated from what would be predicted if all three pathogens assorted independently (Figure 2). Most notably, *B. microti*/*B. burgdorferi* co-infection was 83% greater than predicted if all three pathogens assorted independently (Table 1. Concomitantly, 16% fewer single *B. burgdorferi* infections and 34% fewer single *B. microti* infections were found than would be expected given independent assortment (Table 1). There were 31% fewer *A. phagocytophilum/B. microti* co-infections than expected by chance, and no statistically significant deviations from chance were detected in *A. phagocytophilum/B. burgdorferi* co-infections or single *A. phagocytophilum* infections at α = 0.05 (Table 1). Triple infections were 89% more common than expected by chance, while uninfected ticks were 7% more common (Table 1).

**Table 1:** Predictions of infection prevalence in questing *Ixodes scapularis* nymphal ticks using a permutation analysis assuming independent assortment of all three pathogens, and deviations of observed data from those predictions.

Pathogen or pathogen combination	Mean expected prevalence (%)	2.5% quantile	97.5% quantile	Actual prevalence (%)	*p*-value	Observed: Expected
*A. phagocytophilum* (Ap)	5.18	4.76	5.59	4.78	0.0596	0.92
*B. microti* (Bm)	8.91	8.42	9.41	5.84	**<0.0001**	0.66
*B. burgdorferi* (Bb)	22.99	22.44	23.53	19.32	**<0.0001**	0.84
Ap + Bm	0.83	0.60	1.08	0.57	**0.0476**	0.69
Ap + Bb	2.13	1.76	2.50	2.47	0.0633	1.16
Bm + Bb	3.66	3.21	4.12	6.68	**<0.0001**	1.83
All three pathogens	0.34	0.18	0.53	0.64	**0.0010**	1.89
Uninfected	55.96	55.38	56.55	59.68	**<0.0001**	1.07

Bolded *p-*values are significant at α = 0.05.

Similar patterns of co-infection occurred for both the combined 2011–2012 data and for each year analyzed separately (Tables 1, S1). In 2011 alone, there were no statistically significant deviations from chance involving *A. phagocytophilum* single infections or co-infections (Table S1). In 2012 alone, there were 28% fewer single *A. phagocytophilum* infections than expected by chance, and over three times as many triple infections (though triply infected ticks were rare; Table S1). In both 2011 and 2012, uninfected ticks were 5% and 10% more common than predicted, respectively (Table S1). The directionality of the trends in co-infection did not vary among 2011 alone, 2012 alone, or the two years of data combined.

### Host-collected ticks

**Figure 3:**
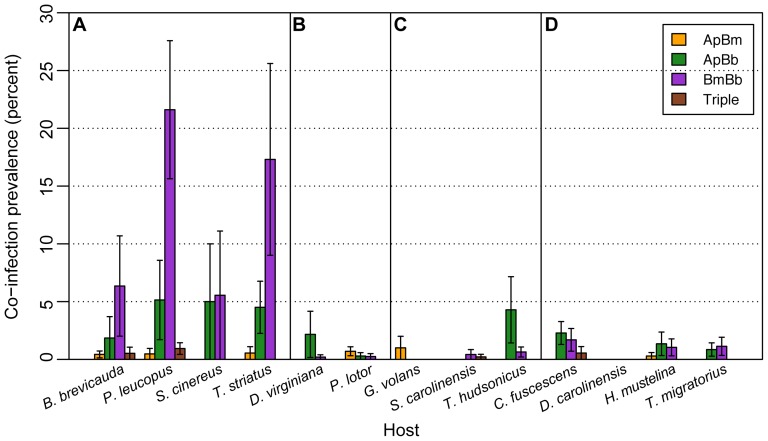
Co-infection prevalence of Anaplasma phagocytophilum, Babesia microti, and Borrelia burgdorferi in wildlife host species groups. Mean co-infection prevalence for Anaplasma phagocytophilum (Ap), Babesia microti (Bm), and Borrelia burgdorferi (Bb) of host-collected Ixodes scapularis ticks fed on (A) small mammal, (B) meso-mammal, (C) sciurid, and (D) bird host species. Each category represents the mean prevalence of each specific co-infection type as opposed to overall prevalence. Error bars show standard error. Note that no co-infections were observed in D. carolinensis.

As with questing nymphs, observed levels of co-infection for host-collected ticks also often deviated from predictions based on independent assortment. However, these patterns differed among wildlife hosts. A similar pattern of greater *B. microti*/*B. burgdorferi* co-infection than expected by chance was also found in host-collected ticks fed on small mammal hosts, but not birds, sciurids, or meso-mammals (Figure 3, Tables 2–5, S2–S3). *B. microti/B. burgdorferi* co-infections were 35% more frequent than predicted in ticks fed on small mammals, but this pattern was not repeated in ticks fed on meso-mammals, sciurids, or birds (Tables 2–5). Similarly, there were 20% fewer single *B. microti* and 9% fewer single *B. burgdorferi* infections than predicted in ticks fed on small mammals (Table 2). When each species was analyzed individually, the same pattern held. *B. microti/B. burgdorferi* co-infections were more common than expected by chance in ticks fed on *B. brevicauda* (nearly three times as many co-infections as expected), *P. leucopus* (27%), and *T. striatus* (28%) (Table S3). In one bird species (*C. fuscescens*) there were 43% fewer *B. microti/B. burgdorferi* co-infections than expected in host-collected ticks, but *B. microti* infection was rare (Tables S2–S3).

**Table 2:** Predictions of infection prevalence in host-collected ticks fed on small mammals using a permutation analysis assuming independent assortment of all three pathogens, and deviations of observed data from those predictions.

Pathogen or pathogen combination	Mean expected prevalence (%)	2.5% quantile	97.5% quantile	Actual prevalence (%)	*p*-value	Observed: Expected
*A. phagocytophilum* (Ap)	5.70	4.81	6.56	7.22	**0.001**	1.27
*B. microti* (Bm)	9.27	8.10	10.39	7.44	**0.002**	0.80
*B. burgdorferi* (Bb)	30.77	29.54	32.06	28.12	**<0.0001**	0.91
Ap + Bm	1.33	0.66	1.97	0.44	**0.009**	0.33
Ap + Bb	3.56	2.74	4.38	3.50	0.900	0.98
Bm + Bb	9.41	8.32	10.50	12.69	**<0.0001**	1.35
All three pathogens	1.11	0.55	1.75	0.55	0.076	0.49
Uninfected	38.84	37.53	40.15	40.04	0.083	1.03

Bolded *p-*values are significant at α = 0.05.

**Table 3:** Predictions of infection prevalence in host-collected ticks fed on meso-mammals using a permutation analysis assuming independent assortment of all three pathogens, and deviations of observed data from those predictions.

Pathogen or pathogen combination	Mean expected prevalence (%)	2.5% quantile	97.5% quantile	Actual prevalence (%)	*p*-value	Observed: Expected
*A. phagocytophilum* (Ap)	2.84	2.37	3.27	2.14	**0.004**	0.76
*B. microti* (Bm)	13.03	12.40	13.53	13.42	0.238	1.03
*B. burgdorferi* (Bb)	3.50	3.04	3.95	3.04	0.067	0.87
Ap + Bm	0.40	0.11	0.79	0.34	0.788	0.84
Ap + Bb	0.13	0	0.34	0.90	**<0.0001**	7.08
Bm + Bb	0.53	0.11	1.01	0.23	0.204	0.43
All three pathogens	0.02	0	0.11	0	>0.9	0
Uninfected	79.56	79.03	80.16	79.93	0.269	1.00

Bolded *p-*values are significant at α = 0.05.

**Table 4:** Predictions of infection prevalence in host-collected ticks fed on sciurids using a permutation analysis assuming independent assortment of all three pathogens, and deviations of observed data from those predictions.

Pathogen or pathogen combination	Mean expected prevalence (%)	2.5% quantile	97.5% quantile	Actual prevalence (%)	*p*-value	Observed: Expected
*A. phagocytophilum* (Ap)	4.30	3.55	4.95	4.33	>0.9	1.01
*B. microti* (Bm)	2.58	2.01	3.09	2.63	>0.9	1.02
*B. burgdorferi* (Bb)	18.98	18.08	19.78	19.17	0.719	1.01
Ap + Bm	0.16	0	0.46	0.15	>0.9	0.97
Ap + Bb	1.22	0.62	1.85	1.08	0.824	0.89
Bm + Bb	0.62	0.15	1.08	0.46	0.579	0.74
All three pathogens	0.04	0	0.31	0.15	0.232	3.85
Uninfected	72.10	71.25	72.95	72.02	>0.9	1.00

Bolded *p-*values are significant at α = 0.05.

**Table 5:** Predictions of infection prevalence in host-collected ticks fed on birds using a permutation analysis assuming independent assortment of all three pathogens, and deviations of observed data from those predictions.

Pathogen or pathogen combination	Mean expected prevalence (%)	2.5% quantile	97.5% quantile	Actual prevalence (%)	*p*-value	Observed: Expected
*A. phagocytophilum* (Ap)	2.03	1.45	2.54	2.18	0.670	1.07
*B. microti* (Bm)	1.02	0.60	1.45	1.09	0.803	1.07
*B. burgdorferi* (Bb)	52.56	51.87	53.33	53.08	0.186	1.01
Ap + Bm	0.05	0	0.24	0.12	0.372	2.21
Ap + Bb	2.08	1.57	2.66	1.69	0.205	0.81
Bm + Bb	1.52	1.09	1.93	1.21	0.222	0.80
All three pathogens	0.07	0	0.24	0.24	0.110	3.48
Uninfected	40.67	39.90	41.35	40.39	0.506	0.99

Bolded *p-*values are significant at α = 0.05.

Several other patterns of co-infection involving *A. phagocytophilum* were observed in ticks fed on different animal groups. In ticks fed on small mammals, there were 27% more single *A. phagocytophilum* infections, and fewer *A. phagocytophilum/B. microti* co-infections and triple infections than expected from independent assortment (Table 2). In ticks fed on meso-mammals, there were seven times more *A. phagocytophilum/B. burgdorferi* co-infections than predicted, and 25% fewer single *A. phagocytophilum* infections (Table 3). In ticks fed on both sciurids and birds, there were no patterns of co-infection that differed significantly from what was expected by chance (Tables 4–5, Table S3).

Infection prevalence at the host level varied among species (Table S4). Individual hosts were considered infected for a given pathogen if at least one tick known to feed on that individual as a larva emerged infected with that pathogen. The highest prevalence of co-infection for all pathogen combinations (including both double and triple infections) was observed in *P. leucopus*.

## Discussion

Patterns of co-infection of both questing nymphs and host-collected ticks deviated from co-infection patterns predicted by independent assortment in several ways. Co-infection of questing nymphal ticks with *Babesia microti* and *Borrelia burgdorferi* occurred more often than expected by chance. This pattern also appeared in small mammal hosts but not other host groups (sciurids, meso-mammals, birds). Co-infection with *A. phagocytophilum* and *B. microti* in questing nymphs was less common than expected given independent assortment, and again this pattern was seen in small mammal hosts but not other host groups.

The positive association between *Babesia microti* and *Borrelia burgdorferi* in both questing nymphs and host-collected ticks fed on small-mammal hosts was the most striking pattern observed. Larval *I. scapularis* ticks that feed on small rodents and shrews are more likely both to acquire infection with each of the three tick-borne pathogens [22]–[24] and to acquire dual infection with *B. microti* and *B. burgdorferi* than ticks feeding on other host groups. The results presented in this study are consistent with regional studies of questing ticks [33] and human infections [2], [7], [8]. Lower resistance and/or higher tolerance of small mammals to *B. microti* and *B. burgdorferi*, leading to correlated reservoir competence, could cause these pathogens to assort together rather than independently. Alternatively, infection with one could facilitate either transmission or proliferation of the other, although such facilitation has not been demonstrated, to our knowledge. High tick loads on small mammals may also facilitate co-infection. Previous studies in this system have shown a correlation between high larval and nymphal burdens in white-footed mice [34]. In theory, animals bitten by more nymphs seem likely to have a heightened risk of obtaining multiple infections through repeated tick exposures. If these same individuals are feeding more larvae, this could further increase the abundance of co-infected nymphs.

Few positive associations occurred between *A. phagocytophilum* and the other two pathogens, with the exception of greater *A. phagocytophilum*/*B. burgdorferi* co-infection in meso-mammal hosts (in particular *D. virginiana*). This is consistent with meta-analyses showing no general pattern of positive association between *A. phagocytophilum* and *B. burgdorferi* in questing *I. scapularis* nymphs [35], [36] and either no facilitation or perhaps some inhibition of transmission of these two pathogens in mouse hosts [16], [21]. Meta-analyses of questing tick infection with these two pathogens have revealed idiosyncratic patterns of co-infection for *B. burgdorferi* and *A. phagocytophilum* at the population level [35], [36]. In *I. scapularis* ticks, the general trend was a lack of significant difference in levels of *B. burgdorferi*/*A. phagocytophilum* co-infection compared to random expectations, although levels of co-infection greater than random expectations occurred more frequently in *Ixodes ricinus* and *Ixodes pacificus* [35], [36]. We observed low levels (4.23%) of *A. phagocytophilum*/*B. burgdorferi* infection in host-collected ticks fed on white-footed mice (Table S3-B). *A. phagocytophilum* infections seem to be transient in wildlife hosts [37], such that nymphal inoculation followed rapidly by larval feeding may be required for transmission. As a consequence, extrinsic factors such as the timing of larval feeding with respect to nymphal inoculation, in addition to factors intrinsic to hosts, might be important in determining host-specific reservoir competence. This might lead to lower variance among hosts in reservoir competence and therefore co-infection. Indeed, data from Hersh et al. [22] and Keesing et al. [23] show that variance among hosts in *A. phagocytophilum* reservoir competence is low compared to variance for *B. microti* or *B. burgdorferi* [24], so that host-specific biases should be more modest. In this dataset, the standard deviation among species in *B. burgdorferi* realized reservoir competence is 32.54%, compared to 9.64% and 3.76% in *B. microti* and *A. phagocytophilum*, respectively.

Rates of co-infection in this study are comparable to other regional studies on *I. scapularis* ticks. Even higher rates of *B. burgdorferi/B. microti* co-infection in questing nymphs have been measured on Nantucket and Nashuon islands [38], albeit with a smaller sample size. In Maryland, rates of co-infection with *B. burgdorferi* and any one of several other pathogens included in the study were higher than expected by chance [39]. Other studies of infection in adult *I. scapularis* ticks have demonstrated co-infection, sometimes at higher levels, [40]–[44] although studies examining adult ticks might not pertain well to estimating disease risk to people given that nymphs transmit most, and adults relatively few, cases of tick-borne diseases [5]. Further, adults may have obtained infections from either or both of their larval and nymphal blood meals.

Overall, we find non-independent assortment of three tickborne pathogens in both questing nymphal ticks and ticks fed on small mammal host species, most notably *B. microti* and *B. burgdorferi*. These results suggest that factors that regulate abundance of these small mammals hosts, including predation and forest fragmentation [45], [46] would likely impact both independent and combined infection prevalence in tick vectors. They also suggest that exposure of human patients with *B. microti* and *B. burgdorferi* should be expected to be a relatively common consequence of single tick bites within endemic zones. As our calculations focus on exposure to multiple pathogens from a single bite, further studies are needed to quantify rates of transmission of multiple pathogens from co-infected nymphs to vertebrate hosts. Similarly, quantification of co-infection of vertebrates from sequential bites requires additional study. Given that co-infection for these two pathogens can exacerbate symptoms and requires distinct treatment [9], medical practitioners should be aware of the tendency for *B. microti* and *B. burgdorferi* to co-occur when diagnosing and treating tickborne illness.

## Supporting Information

Figure S1 **Map of locations sampled for questing nymphal ticks across Dutchess County, New York.**
(DOC)

Figure S2 **Extent of co-infection with** ***Anaplasma phagocytophilum, Babesia microti*** **, and** ***Borrelia burgdorferi*** **in questing nymphal** ***Ixodes scapularis*** **ticks in 2011, 2012, and both years combined.**
(DOC)

Table S1 **Permutation-based predictions of infection prevalence in questing** ***Ixodes scapularis*** **nymphal ticks assuming independent assortment of all three pathogens, and deviations of observed data from those predictions for 2011 and 2012 questing nymphs.**
(DOC)

Table S2 **Sample sizes and mean levels of infection and co-infection for host-collected ticks for** ***Anaplasma phagocytophilum, Babesia microti*** **, and** ***Borrelia burgdorferi*** **.**
(DOC)

Table S3 **Permutation-based predictions of infection prevalence in host-collected** ***Ixodes scapularis*** **ticks assuming independent assortment of all three pathogens, and deviations of observed data from those predictions for individual host species with >100 ticks sampled.**
(DOC)

Table S4 **Levels of infection and co-infection for individual wildlife hosts for** ***Anaplasma phagocytophilum*** **(Ap),** ***Babesia microti*** **(Bm), and** ***Borrelia burgdorferi*** **.**
(DOC)
