# Supplementary figures and images for: Co-Infection of Blacklegged Ticks with Babesia microti and Borrelia burgdorferi Is Higher than Expected and Acquired from Small Mammal Hosts
Source: PLoS One. 2014 Jun 18;9(6):e99348. doi: 10.1371/journal.pone.0099348 (PMC4062422; doi:10.1371/journal.pone.0099348)

**Figure S1.** Map of locations sampled for questing nymphal ticks across Dutchess County, New York.


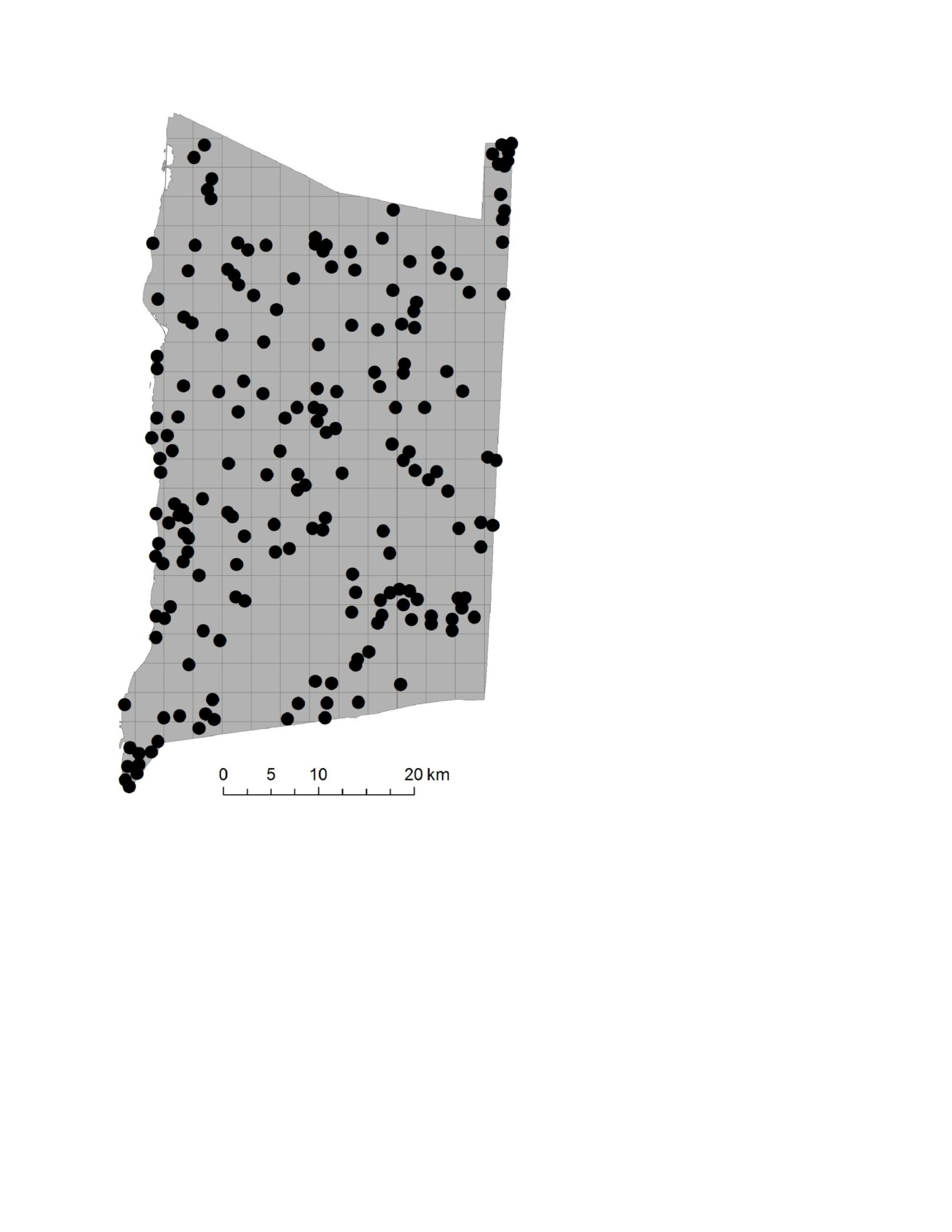

Supplement: Figure S1 — Map of locations sampled for questing nymphal ticks across Dutchess County, New York. (DOC) [file pone.0099348.s001.doc]
