# Supplementary material for: Co-Infection of Blacklegged Ticks with Babesia microti and Borrelia burgdorferi Is Higher than Expected and Acquired from Small Mammal Hosts
Source: PLoS One. 2014 Jun 18;9(6):e99348. doi: 10.1371/journal.pone.0099348 (PMC4062422; doi:10.1371/journal.pone.0099348)

**Figure S2.** Extent of co-infection with *Anaplasma phagocytophilum* (Ap)*, Babesia microti* (Bm), and *Borrelia burgdorferi* (Bb) in questing nymphal *Ixodes scapularis* ticks in 2011, 2012, and both years combined. Each category represents mean overall prevalence as opposed to the prevalence of each specific infection type, as in Figure 1.


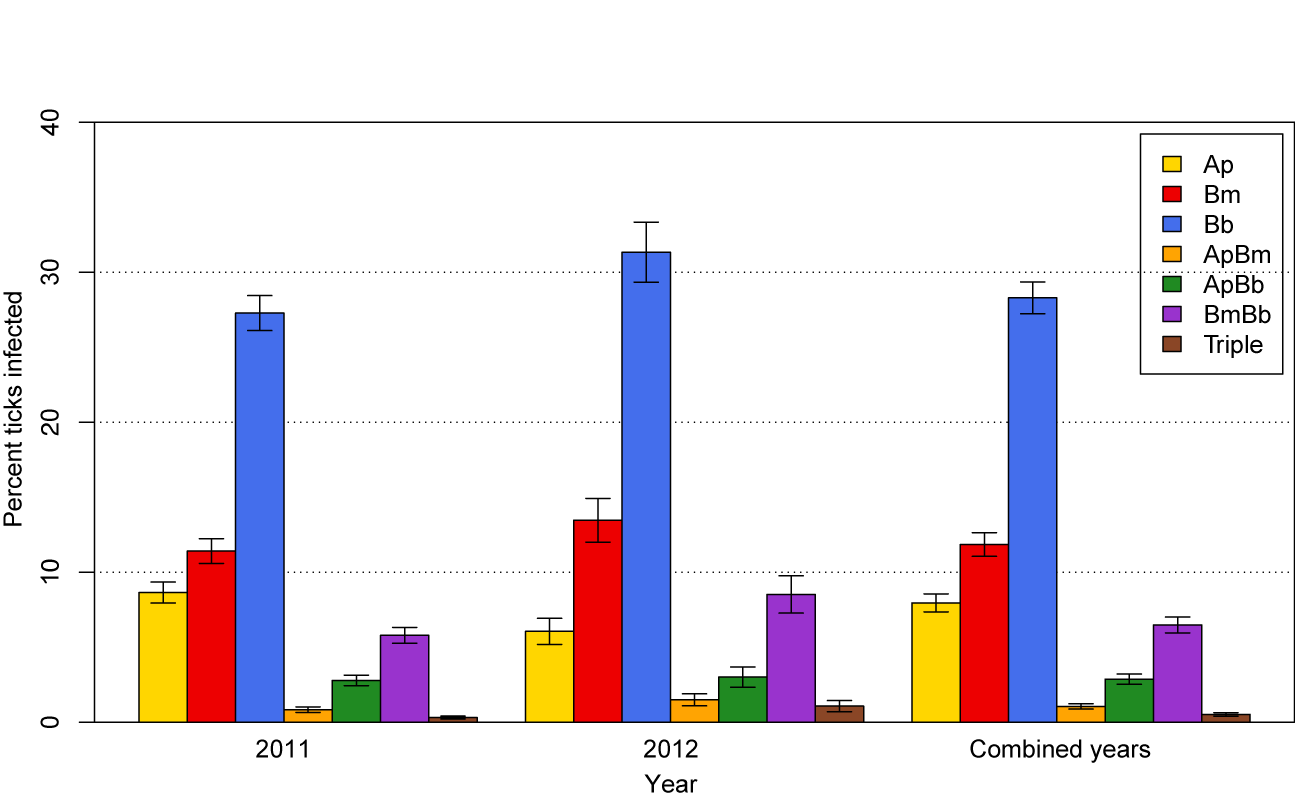

Supplement: Figure S2 — Extent of co-infection with Anaplasma phagocytophilum, Babesia microti , and Borrelia burgdorferi in questing nymphal Ixodes scapularis ticks in 2011, 2012, and both years combined. (DOC) [file pone.0099348.s002.doc]
