# Supplementary material for: Co-Infection of Blacklegged Ticks with Babesia microti and Borrelia burgdorferi Is Higher than Expected and Acquired from Small Mammal Hosts
Source: PLoS One. 2014 Jun 18;9(6):e99348. doi: 10.1371/journal.pone.0099348 (PMC4062422; doi:10.1371/journal.pone.0099348)
Supplement: Table S1 — Permutation-based predictions of infection prevalence in questing Ixodes scapularis nymphal ticks assuming independent assortment of all three pathogens, and deviations of observed data from those predictions for 2011 and 2012 questing nymphs. (DOC) [file pone.0099348.s003.doc]

**Table S1.** Permutation-based predictions of infection prevalence in questing *Ixodes scapularis* nymphal ticks assuming independent assortment of all three pathogens, and deviations of observed data from those predictions for 2011 (Table S1-A) and 2012 (Table S1-B) questing nymphs.

**Table S1-A.** 2011.

| **Pathogen or pathogen combination** | **Mean expected prevalence (%)** | **2.5% quantile** | **97.5% quantile** | **Actual prevalence (%)** | ***p*-value** | **Observed: Expected** |
| --- | --- | --- | --- | --- | --- | --- |
| *A. phagocytophilum* (Ap) | 5.81 | 5.31 | 6.28 | 5.59 | 0.4128 | 0.96 |
| *B. microti* (Bm) | 8.28 | 7.73 | 8.83 | 5.68 | **<0.0001** | 0.69 |
| *B. burgdorferi* (Bb) | 22.03 | 21.39 | 22.64 | 18.75 | **<0.0001** | 0.85 |
| Ap + Bm | 0.84 | 0.57 | 1.13 | 0.57 | 0.0738 | 0.67 |
| Ap + Bb | 2.23 | 1.82 | 2.67 | 2.64 | 0.0751 | 1.18 |
| Bm + Bb | 3.18 | 2.67 | 3.71 | 5.97 | **<0.0001** | 1.87 |
| All three pathogens | 0.32 | 0.16 | 0.53 | 0.41 | 0.41536 | 1.27 |
| Uninfected | 57.31 | 56.63 | 57.98 | 60.40 | **<0.0001** | 1.05 |

**Table S1-B.** 2012.

| **Pathogen or pathogen combination** | **Mean expected prevalence (%)** | **2.5% quantile** | **97.5% quantile** | **Actual prevalence (%)** | ***p*-value** | **Observed: Expected** |
| --- | --- | --- | --- | --- | --- | --- |
| *A. phagocytophilum* (Ap) | 3.64 | 2.96 | 4.31 | 2.62 | **0.0045** | 0.72 |
| *B. microti* (Bm) | 10.51 | 9.46 | 11.57 | 6.25 | **<0.0001** | 0.59 |
| *B. burgdorferi* (Bb) | 25.52 | 24.41 | 26.60 | 20.86 | **<0.0001** | 0.82 |
| Ap + Bm | 0.73 | 0.34 | 1.18 | 0.59 | 0.5855 | 0.81 |
| Ap + Bb | 1.78 | 1.18 | 2.45 | 2.03 | 0.5097 | 1.14 |
| Bm + Bb | 5.12 | 4.14 | 6.08 | 8.61 | **<0.0001** | 1.68 |
| All three pathogens | 0.36 | 0.08 | 0.68 | 1.27 | **<0.0001** | 3.55 |
| Uninfected | 52.35 | 51.18 | 53.55 | 57.77 | **<0.0001** | 1.10 |
