# Supplementary material for: Co-Infection of Blacklegged Ticks with Babesia microti and Borrelia burgdorferi Is Higher than Expected and Acquired from Small Mammal Hosts
Source: PLoS One. 2014 Jun 18;9(6):e99348. doi: 10.1371/journal.pone.0099348 (PMC4062422; doi:10.1371/journal.pone.0099348)
Supplement: Table S2 — Sample sizes and mean levels of infection and co-infection for host-collected ticks for Anaplasma phagocytophilum, Babesia microti , and Borrelia burgdorferi . (DOC) [file pone.0099348.s004.doc]

**Table S2.** Sample sizes and mean levels of infection and co-infection for larvae fed on specific wildlife host species for *Anaplasma phagocytophilum* (Ap)*, Babesia microti* (Bm)*,* and *Borrelia burgdorferi* (Bb). Mean levels of infection and co-infection represent the average percentage of ticks found on an individual of a given host species with that particular infection status.

| **Species** | **Group** | **Total hosts sampled** | **Total ticks sampled** | **Mean ticks/host (SD)** | **Mean % uninfected ticks** | **Mean % Ap only** | **Mean % Bm only** | **Mean % Bb only** | **Mean % Ap Bm** | **Mean % Ap Bb** | **Mean % Bm Bb** | **Mean % Ap Bm Bb** |
| --- | --- | --- | --- | --- | --- | --- | --- | --- | --- | --- | --- | --- |
| *B. brevicauda* | small mammals | 21 | 406 | 19.3 (4.64) | 66.79 | 10.73 | 8.98 | 4.32 | 0.43 | 1.85 | 6.35 | 0.53 |
| *P. leucopus* | small mammals | 14 | 260 | 18.6 (4.18) | 18.36 | 6.25 | 3.84 | 43.38 | 0.48 | 5.15 | 21.61 | 0.94 |
| *S. cinereus* | small mammals | 6 | 41 | 6.8 (2.56) | 25.56 | 2.78 | 17.78 | 43.33 | 0 | 5 | 5.56 | 0 |
| *T. striatus* | small mammals | 13 | 207 | 15.9 (4.96) | 19.22 | 3.63 | 1.55 | 53.23 | 0.55 | 4.51 | 17.31 | 0 |
| *D. virginiana* | meso-mammals | 25 | 501 | 20.0 (4.69) | 85.51 | 2.64 | 6.62 | 2.87 | 0 | 2.17 | 0.20 | 0 |
| *P. lotor* | meso-mammals | 20 | 386 | 19.3 (3.53) | 73.03 | 1.72 | 20.98 | 3.02 | 0.71 | 0.29 | 0.25 | 0 |
| *G. volans* | sciurids | 4 | 59 | 14.8 (9.67) | 87.07 | 6.57 | 5.36 | 0 | 1.00 | 0 | 0 | 0 |
| *S. carolinensis* | sciurids | 18 | 333 | 18.5 (4.42) | 82.67 | 4.16 | 2.19 | 10.33 | 0 | 0 | 0.43 | 0.22 |
| *T. hudsonicus* | sciurids | 13 | 255 | 19.6 (4.56) | 53.79 | 5.12 | 2.04 | 34.11 | 0 | 4.29 | 0.64 | 0 |
| *C. fuscescens* | birds | 15 | 308 | 20.5 (4.87) | 18.40 | 1.16 | 1.48 | 74.42 | 0 | 2.29 | 1.69 | 0.56 |
| *D. carolinensis* | birds | 6 | 93 | 15.5 (6.89) | 95.83 | 3.33 | 0 | 0.83 | 0 | 0 | 0 | 0 |
| *H. mustelina* | birds | 14 | 234 | 16.7 (4.63) | 69.88 | 2.92 | 2.41 | 22.09 | 0.30 | 1.35 | 1.05 | 0 |
| *T. migratorius* | birds | 12 | 192 | 16.0 (5.86) | 18.72 | 0.88 | 0 | 78.41 | 0 | 0.86 | 1.13 | 0 |
