# Supplementary material for: Co-Infection of Blacklegged Ticks with Babesia microti and Borrelia burgdorferi Is Higher than Expected and Acquired from Small Mammal Hosts
Source: PLoS One. 2014 Jun 18;9(6):e99348. doi: 10.1371/journal.pone.0099348 (PMC4062422; doi:10.1371/journal.pone.0099348)
Supplement: Table S3 — Permutation-based predictions of infection prevalence in host-collected Ixodes scapularis ticks assuming independent assortment of all three pathogens, and deviations of observed data from those predictions for individual host species with >100 ticks sampled. (DOC) [file pone.0099348.s005.doc]

**Table S3.** Permutation-based predictions of infection prevalence in newly fed *Ixodes scapularis* larvae assuming independent assortment of all three pathogens, and deviations of observed data from those predictions for individual host species with >100 ticks sampled. Hosts include small mammal species *Blarina brevicauda* (Table S3-A)*, Peromyscus leucopus* (Table S3-B) and *Tamias striatus* (Table S3-C); meso-mammal species *Didelphis virginiana* (Table S3-D) and *Procyon lotor* (Table S3-E); sciurid species *Sciurus carolinensis* (Table S3-F) and *Tamiasciurus hudsonicus* (Table S3-G); and bird species *Catharus fuscescens* (Table S3-H)*, Hylocichla mustelina* (Table S3-I), and *Turdus migratorius* (Table S3-J). Small mammal species *Sorex cinereus,* sciurid species *Glaucomys volans,* and bird species *Dumetella carolinensis* were not sampled sufficiently to be included in this analysis (<100 ticks/species).

***Table S3-A.*** *Blarina brevicauda (short-tailed shrew, n=406)*

| **Pathogen or pathogen combination** | **Mean expected prevalence (%)** | **2.5% quantile** | **97.5% quantile** | **Actual prevalence (%)** | ***p*-value** | **Observed: Expected** |
| --- | --- | --- | --- | --- | --- | --- |
| *A. phagocytophilum* (Ap) | 10.15 | 8.62 | 11.58 | 11.08 | 0.259 | 1.09 |
| *B. microti* (Bm) | 12.77 | 11.08 | 14.29 | 10.84 | **0.020** | 0.85 |
| *B. burgdorferi* (Bb) | 8.31 | 6.90 | 9.61 | 4.43 | **<0.0001** | 0.53 |
| Ap + Bm | 2.04 | 0.99 | 3.20 | 0.49 | **0.014** | 0.24 |
| Ap + Bb | 1.33 | 0.49 | 2.46 | 1.72 | 0.467 | 1.30 |
| Bm + Bb | 1.67 | 0.74 | 2.71 | 4.93 | **<0.0001** | 2.95 |
| All three pathogens | 0.27 | 0 | 0.74 | 0.49 | 0.624 | 1.83 |
| Uninfected | 63.46 | 61.82 | 65.27 | 66.01 | **0.007** | 1.04 |

***Table S3-B.*** *Peromyscus leucopus (white-footed mouse, n=260)*

| **Pathogen or pathogen combination** | **Mean expected prevalence (%)** | **2.5% quantile** | **97.5% quantile** | **Actual prevalence (%)** | ***p*-value** | **Observed: Expected** |
| --- | --- | --- | --- | --- | --- | --- |
| *A. phagocytophilum* (Ap) | 2.41 | 0.77 | 4.23 | 5.77 | **<0.001** | 2.39 |
| *B. microti* (Bm) | 7.36 | 5.00 | 10.00 | 4.23 | 0.014 | 0.57 |
| *B. burgdorferi* (Bb) | 44.78 | 41.92 | 47.69 | 42.69 | 0.200 | 0.95 |
| Ap + Bm | 0.96 | 0 | 2.31 | 0.38 | 0.501 | 0.40 |
| Ap + Bb | 5.85 | 3.85 | 7.69 | 4.23 | 0.126 | 0.72 |
| Bm + Bb | 17.82 | 15.00 | 20.38 | 22.69 | **<0.001** | 1.27 |
| All three pathogens | 2.32 | 0.77 | 3.85 | 1.15 | 0.164 | 0.50 |
| Uninfected | 18.50 | 15.77 | 21.15 | 18.85 | 0.890 | 1.02 |

**Table S3-C.** *Tamias striatus* (Eastern chipmunk, n=207)

| **Pathogen or pathogen combination** | **Mean expected prevalence (%)** | **2.5% quantile** | **97.5% quantile** | **Actual prevalence (%)** | ***p*-value** | **Observed: Expected** |
| --- | --- | --- | --- | --- | --- | --- |
| *A. phagocytophilum* (Ap) | 1.57 | 0.48 | 3.38 | 2.42 | 0.336 | 1.54 |
| *B. microti* (Bm) | 4.09 | 1.93 | 6.28 | 1.45 | **0.030** | 0.35 |
| *B. burgdorferi* (Bb) | 56.86 | 54.11 | 59.42 | 54.11 | 0.059 | 0.95 |
| Ap + Bm | 0.37 | 0 | 1.45 | 0.48 | >0.9 | 1.32 |
| Ap + Bb | 5.09 | 3.38 | 6.76 | 5.31 | >0.9 | 1.04 |
| Bm + Bb | 13.20 | 10.63 | 15.46 | 16.91 | **0.003** | 1.28 |
| All three pathogens | 1.19 | 0 | 2.42 | 0 | 0.146 | 0 |
| Uninfected | 17.64 | 14.98 | 20.29 | 19.32 | 0.266 | 1.10 |

**Table S3-D.** *Didelphis virginiana* (Virginia opossum, n=501)

| **Pathogen or pathogen combination** | **Mean expected prevalence (%)** | **2.5% quantile** | **97.5% quantile** | **Actual prevalence (%)** | ***p*-value** | **Observed: Expected** |
| --- | --- | --- | --- | --- | --- | --- |
| *A. phagocytophilum* (Ap) | 3.54 | 2.99 | 3.99 | 2.59 | **0.004** | 0.73 |
| *B. microti* (Bm) | 6.40 | 5.79 | 6.99 | 6.79 | 0.345 | 1.06 |
| *B. burgdorferi* (Bb) | 4.10 | 3.39 | 4.59 | 2.99 | **0.001** | 0.73 |
| Ap + Bm | 0.27 | 0 | 0.80 | 0 | 0.385 | 0 |
| Ap + Bb | 0.17 | 0 | 0.60 | 1.40 | **<0.0001** | 8.22 |
| Bm + Bb | 0.31 | 0 | 0.80 | 0.20 | 0.731 | 0.65 |
| All three pathogens | 0.01 | 0 | 0.20 | 0 | >0.9 | 0 |
| Uninfected | 85.20 | 84.63 | 86.03 | 86.03 | **0.030** | 1.01 |

**Table S3-E.** *Procyon lotor* (raccoon, n=386)

| **Pathogen or pathogen combination** | **Mean expected prevalence (%)** | **2.5% quantile** | **97.5% quantile** | **Actual prevalence (%)** | ***p*-value** | **Observed: Expected** |
| --- | --- | --- | --- | --- | --- | --- |
| *A. phagocytophilum* (Ap) | 1.92 | 1.30 | 2.59 | 1.55 | 0.464 | 0.81 |
| *B. microti* (Bm) | 21.64 | 20.47 | 22.54 | 22.02 | 0.618 | 1.02 |
| *B. burgdorferi* (Bb) | 2.72 | 1.81 | 3.37 | 3.11 | 0.382 | 1.14 |
| Ap + Bm | 0.57 | 0 | 1.30 | 0.78 | 0.698 | 1.35 |
| Ap + Bb | 0.07 | 0 | 0.52 | 0.26 | 0.248 | 3.59 |
| Bm + Bb | 0.82 | 0 | 1.55 | 0.26 | 0.206 | 0.32 |
| All three pathogens | 0.02 | 0 | 0.26 | 0 | >0.9 | 0 |
| Uninfected | 72.23 | 71.24 | 73.32 | 72.02 | 0.809 | 1.00 |

**Table S3-F.** *Sciurus carolinensis* (Eastern gray squirrel, n=333)

| **Pathogen or pathogen combination** | **Mean expected prevalence (%)** | **2.5% quantile** | **97.5% quantile** | **Actual prevalence (%)** | ***p*-value** | **Observed: Expected** |
| --- | --- | --- | --- | --- | --- | --- |
| *A. phagocytophilum* (Ap) | 3.84 | 3.00 | 4.50 | 4.20 | 0.488 | 1.09 |
| *B. microti* (Bm) | 2.78 | 2.10 | 3.30 | 2.70 | >0.9 | 0.97 |
| *B. burgdorferi* (Bb) | 10.81 | 9.91 | 11.71 | 11.11 | 0.751 | 1.03 |
| Ap + Bm | 0.13 | 0 | 0.60 | 0 | >0.9 | 0 |
| Ap + Bb | 0.51 | 0 | 1.20 | 0 | 0.234 | 0 |
| Bm + Bb | 0.37 | 0 | 1.20 | 0.30 | >0.9 | 0.81 |
| All three pathogens | 0.02 | 0 | 0.30 | 0.30 | 0.058 | 16.91 |
| Uninfected | 81.53 | 80.78 | 82.58 | 81.38 | >0.9 | 1.00 |

**Table S3-G.** *Tamiasciurus hudsonicus* (American red squirrel, n=255)

| **Pathogen or pathogen combination** | **Mean expected prevalence (%)** | **2.5% quantile** | **97.5% quantile** | **Actual prevalence (%)** | ***p*-value** | **Observed: Expected** |
| --- | --- | --- | --- | --- | --- | --- |
| *A. phagocytophilum* (Ap) | 4.03 | 2.35 | 5.49 | 3.92 | >0.9 | 0.97 |
| *B. microti* (Bm) | 1.83 | 0.78 | 2.75 | 2.35 | 0.476 | 1.29 |
| *B. burgdorferi* (Bb) | 34.03 | 32.16 | 35.69 | 34.12 | >0.9 | 1.00 |
| Ap + Bm | 0.13 | 0 | 0.78 | 0 | >0.9 | 0 |
| Ap + Bb | 2.43 | 1.18 | 3.92 | 2.75 | 0.795 | 1.13 |
| Bm + Bb | 1.10 | 0 | 1.96 | 0.78 | 0.717 | 0.71 |
| All three pathogens | 0.08 | 0 | 0.39 | 0 | >0.9 | 0 |
| Uninfected | 56.37 | 54.51 | 58.04 | 56.08 | 0.831 | 0.99 |

**Table S3-H.** *Catharus fuscescens* (veery, n=308)

| **Pathogen or pathogen combination** | **Mean expected prevalence (%)** | **2.5% quantile** | **97.5% quantile** | **Actual prevalence (%)** | ***p*-value** | **Observed: Expected** |
| --- | --- | --- | --- | --- | --- | --- |
| *A. phagocytophilum* (Ap) | 1.02 | 0 | 1.95 | 1.30 | 0.743 | 1.27 |
| *B. microti* (Bm) | 0.87 | 0 | 1.95 | 1.62 | 0.150 | 1.86 |
| *B. burgdorferi* (Bb) | 70.29 | 69.16 | 71.75 | 71.75 | **0.038** | 1.02 |
| Ap + Bm | 0.04 | 0 | 0.32 | 0 | >0.9 | 0 |
| Ap + Bb | 3.35 | 2.27 | 4.22 | 2.60 | 0.209 | 0.78 |
| Bm + Bb | 2.85 | 1.95 | 3.57 | 1.62 | **0.019** | 0.57 |
| All three pathogens | 0.13 | 0 | 0.65 | 0.65 | 0.060 | 4.82 |
| Uninfected | 21.44 | 20.13 | 22.73 | 20.45 | 0.148 | 0.95 |

**Table S3-I.** *Hylocichla mustelina* (wood thrush, n=234)

| **Pathogen or pathogen combination** | **Mean expected prevalence (%)** | **2.5% quantile** | **97.5% quantile** | **Actual prevalence (%)** | ***p*-value** | **Observed: Expected** |
| --- | --- | --- | --- | --- | --- | --- |
| *A. phagocytophilum* (Ap) | 3.88 | 2.56 | 5.13 | 3.42 | 0.539 | 0.88 |
| *B. microti* (Bm) | 2.33 | 1.28 | 3.42 | 1.71 | 0.440 | 0.73 |
| *B. burgdorferi* (Bb) | 25.34 | 23.50 | 26.92 | 24.79 | 0.609 | 0.98 |
| Ap + Bm | 0.14 | 0 | 0.85 | 0.43 | 0.282 | 3.12 |
| Ap + Bb | 1.49 | 0.43 | 2.99 | 1.71 | 0.751 | 1.15 |
| Bm + Bb | 0.89 | 0 | 2.14 | 1.28 | 0.683 | 1.43 |
| All three pathogens | 0.05 | 0 | 0.43 | 0 | >0.9 | 0 |
| Uninfected | 65.87 | 64.10 | 67.52 | 66.67 | 0.454 | 1.01 |

**Table S3-J.** *Turdus migratorius* (American robin, n=192)

| **Pathogen or pathogen combination** | **Mean expected prevalence (%)** | **2.5% quantile** | **97.5% quantile** | **Actual prevalence (%)** | ***p*-value** | **Observed: Expected** |
| --- | --- | --- | --- | --- | --- | --- |
| *A. phagocytophilum* (Ap) | 0.31 | 0 | 1.04 | 1.04 | 0.106 | 3.36 |
| *B. microti* (Bm) | 0.15 | 0 | 0.52 | 0 | >0.9 | 0 |
| *B. burgdorferi* (Bb) | 82.26 | 81.77 | 83.33 | 82.81 | 0.239 | 1.01 |
| Ap + Bm | 0.003 | 0 | 0* | 0 | >0.9 | 0 |
| Ap + Bb | 1.75 | 1.04 | 2.08 | 1.04 | 0.120 | 0.59 |
| Bm + Bb | 0.87 | 0 | 1.04 | 1.04 | >0.9 | 1.20 |
| All three pathogens | 0.02 | 0 | 0.52 | 0 | >0.9 | 0 |
| Uninfected | 14.64 | 13.54 | 15.10 | 14.06 | 0.222 | 0.96 |

*For this combination, only 0.66% of all permuted samples were not zero.
