# Supplementary material for: Co-Infection of Blacklegged Ticks with Babesia microti and Borrelia burgdorferi Is Higher than Expected and Acquired from Small Mammal Hosts
Source: PLoS One. 2014 Jun 18;9(6):e99348. doi: 10.1371/journal.pone.0099348 (PMC4062422; doi:10.1371/journal.pone.0099348)
Supplement: Table S4 — Levels of infection and co-infection for individual wildlife hosts for Anaplasma phagocytophilum (Ap), Babesia microti (Bm), and Borrelia burgdorferi . (DOC) [file pone.0099348.s006.doc]

**Table S4.** Sample sizes and levels of infection and co-infection for hosts for *Anaplasma phagocytophilum* (Ap)*, Babesia microti* (Bm)*,* and *Borrelia burgdorferi* (Bb). Host infection status was considered positive for each pathogen if at least one fed larva was found to be infected with that pathogen.

| **Species** | **Group** | **Total hosts sampled** | **Number Ap infected (%)** | **Number Bm infected (%)** | **Number Bb infected (%)** | **Number Ap/Bm co-infected (%)** | **Number Ap/Bb co-infected (%)** | **Number Bm/Bb co-infected (%)** | **Number Ap/Bm/Bb co-infected (%)** |
| --- | --- | --- | --- | --- | --- | --- | --- | --- | --- |
| *B. brevicauda* | small mammals | 21 | 12 (57.1) | 11 (52.4) | 14 (66.7) | 7 (33.3) | 8 (38.1) | 8 (38.1) | 5 (23.8) |
| *P. leucopus* | small mammals | 14 | 8 (57.1) | 10 (71.4) | 13 (92.9) | 5 (35.7) | 7 (50.0) | 10 (71.4) | 5 (35.7) |
| *S. cinereus* | small mammals | 6 | 2 (33.3) | 2 (33.3) | 4 (66.7) | 0 (0) | 1 (16.7) | 1 (16.7) | 0 (0) |
| *T. striatus* | small mammals | 13 | 6 (46.2) | 6 (46.2) | 13 (100) | 3 (23.1) | 6 (46.2) | 6 (46.2) | 3 (23.1) |
| *D. virginiana* | meso-mammals | 25 | 9 (36.0) | 11 (44.0) | 8 (32.0) | 3 (12.0) | 2 (8.0) | 1 (4.0) | 0 (0) |
| *P. lotor* | meso-mammals | 20 | 7 (35.0) | 16 (80.0) | 6 (30.0) | 6 (30.0) | 2 (10.0) | 5 (25.0) | 2 (10) |
| *G. volans* | sciurids | 4 | 2 (50.0) | 3 (75.0) | 0 (0) | 1 (25.0) | 0 (0) | 0 (0) | 0 (0) |
| *S. carolinensis* | sciurids | 18 | 12 (66.7) | 6 (33.3) | 9 (50.0) | 5 (27.8) | 8 (44.4) | 4 (22.2) | 4 (22.2) |
| *T. hudsonicus* | sciurids | 13 | 7 (53.8) | 5 (38.5) | 10 (76.9) | 3 (23.1) | 6 (46.2) | 3 (23.1) | 2 (15.4) |
| *C. fuscescens* | birds | 15 | 7 (46.7) | 6 (40.0) | 13 (86.7) | 4 (26.7) | 5 (33.3) | 4 (26.7) | 2 (13.3) |
| *D. carolinensis* | birds | 6 | 1 (16.7) | 0 (0) | 1 (16.7) | 0 (0) | 1 (16.7) | 0 (0) | 0 (0) |
| *H. mustelina* | birds | 14 | 5 (35.7) | 7 (50.0) | 8 (57.1) | 3 (21.4) | 2 (14.3) | 2 (14.3) | 1 (7.1) |
| *T. migratorius* | birds | 12 | 3 (25.0) | 2 (16.7) | 12 (100) | 1 (8.3) | 3 (25.0) | 2 (16.7) | 1 (8.3) |
